# Myeloid-Derived Suppressor Cells (MDSCs) in Cardiovascular Risk of Obesity-Associated Diabetes

**DOI:** 10.3390/ijms27177872

**Published:** 2026-09-03

**Authors:** Rocío Flores-Campos, Lourdes Hontecillas-Prieto, Daniel J. García-Domínguez, Antonio Fernández-Suárez, Iker Egusquiza-Lasuen, Antonio Pérez, Josep Ribalta, Juan Pedro-Botet, Víctor Sánchez-Margalet, on behalf of the Immunology Group of the Spanish Society of Laboratory Medicine (SEMEDLAB), the Cardiovascular Disease Group of the Spanish Diabetes Society (SED)

**Affiliations:** 1 Department of Medical Biochemistry and Molecular Biology and Immunology, School of Medicine, University of Seville, 41009 Seville, Spain; 2 Laboratory Medicine Service, Virgen Macarena University Hospital, University of Seville, 41009 Seville, Spain; 3 Institute of Biomedicine of Seville, Virgen Macarena/Virgen Rocio University Hospital, CSIC, University of Seville, 41004 Seville, Spain; 4 Immunology Group of the Spanish Society of Laboratory Medicine (SEMEDLAB), 28010 Madrid, Spain; 5 Servicio Andaluz de Salud, UGC Laboratorios, Hospital Universitario San Agustín, 23700 Linares, Spain; 6 Osakidetza Basque Health Service, Department of Laboratory, Araba University Hospital, 01009 Vitoria-Gasteiz, Spain; 7 Cardiovascular Risk Group of the Spanish Society of Dibetes (SED), 28002 Madrid, Spain; 8 Centro de Investigación Biomédica en Red de Diabetes y Enfermedades Metabólicas Asociadas (CIBERDEM), Instituto de Salud Carlos III (ISCIII), 28029 Madrid, Spain; 9 Servicio de Endocrinología y Nutrición, Hospital de la Santa Creu i Sant Pau, IIB Sant Pau, Universitat Autònoma de Barcelona, 08193 Barcelona, Spain; 10 Departament de Medicina i Cirurgia, Unitat de Recerca en Lípids i Arteriosclerosi (URLA), Universitat Rovira i Virgili, 43204 Reus, Spain; 11 Institut d’Investigació Sanitària Pere Virgili (IISPV), 43204 Reus, Spain; 12 Hospital del Mar, Unidad de Lípidos y Riesgo Vascular, 08003 Barcelona, Spain; 13 Departamento de Medicina, Universidad Autónoma de Barcelona, 08193 Barcelona, Spain

**Keywords:** myeloid-derived suppressor cells (MDSCs), immunometabolism, obesity-induced inflammation, Type 2 diabetes mellitus, cardiovascular disease, atherosclerosis

## Abstract

The dysregulation of immune homeostasis and the persistence of chronic, low-grade inflammation represent core pathophysiological drivers governing the onset and progression of metabolic and cardiovascular disorders. Myeloid-derived suppressor cells (MDSCs) comprise a highly heterogeneous population of immature myeloid cells that are rapidly mobilized from the bone marrow in response to biological stressors, such as sustained tissue injury and chronic inflammatory signaling. Historically evaluated within oncology and infectious disease frameworks, accumulating evidence highlights the pivotal, context-dependent roles of MDSCs in cardiometabolic environments. In conditions of obesity and type 2 diabetes (T2D), MDSCs undergo substantial expansion and accumulation within peripheral tissues, including adipose tissue and the liver. Functionally, MDSCs act as dynamic immunoregulators that can either buffer metabolic inflammation, via the secretion of interleukin-10 (IL-10), transforming growth factor-beta (TGF-β), and nitric oxide (NO) or, conversely, reflect and exacerbate disease progression depending on specific cellular subsets, glycemic control, and tissue localization. Within the vascular system, specific monocytic MDSC subsets demonstrate significant atheroprotective and cardioprotective capabilities by dampening Th1/Th17-mediated arterial wall inflammation, stabilizing atherosclerotic plaques, and preserving cardiac structural architecture during heart failure. However, phenotypic and functional heterogeneity poses a critical clinical challenge, as certain persistent or altered subsets correlate with heightened cardiovascular risk and adverse cerebrovascular outcomes. This review delivers a comprehensive synthesis of contemporary insights into MDSC biology at the intersection of obesity-associated diabetes and cardiovascular disease, characterizing their dualistic mechanisms, distinguishing preclinical causal mechanisms from human observational data. Moreover, we have established a comprehensive framework to reconcile the conflicting protective versus detrimental effects of MDSCs. And finally, we have also evaluated their potential clinical utility as candidate biomarkers, delineating the safety imperatives essential for translating MDSC-targeted therapeutic interventions into clinical practice.

## 1. Introduction

Immunological homeostasis requires a meticulously balanced state in which the host immune system successfully defends against pathogens and tissue injury while simultaneously preventing aberrant, self-destructive inflammatory responses [1]. When genetic or environmental factors disrupt this equilibrium, a state of chronic, low-grade systemic inflammation frequently emerges, driving the pathogenesis of interrelated metabolic conditions, such as obesity and type 2 diabetes (T2D) and subsequent cardiovascular disease (CVD) [2,3,4,5]. Over the past decade, myeloid-derived suppressor cells (MDSCs) have emerged as essential frontline orchestrators within this inflammatory landscape [6,7,8]. This review provides a unique synthesis focused on the integrated obesity–T2D–CVD continuum incorporating recent findings. Crucially, rather than presenting sequential literature summaries, this manuscript establishes a comprehensive framework to mechanistically reconcile the paradoxical protective versus detrimental roles of MDSCs across distinct disease stages and tissue environments.

MDSCs originate from immature myeloid progenitors in the bone marrow. Under standard physiological conditions, these immature cells follow a highly coordinated differentiation pathway to mature into functional dendritic cells (DCs), macrophages, or granulocytes [9]. However, in pathological settings characterized by persistent inflammatory cytokines and metabolic stress, this normal differentiation cascade is severely disrupted. The altered tissue microenvironment—enriched with specific growth factors and stress signals—impedes regular myeloid maturation, culminating in the systemic mobilization, expansion, and accumulation of functionally active MDSCs endowed with potent immunosuppressive capabilities [10,11,12,13,14,15].

Physiologically and immunologically, MDSCs exert profound suppressive pressure on both adaptive immune components (predominantly $CD4^{+}$ and $CD8^{+}$ T lymphocytes, as well as B cells) and innate effectors (including natural killer [NK] cells, DCs, and pro-inflammatory M1-type macrophages) [16,17,18,19,20]. Furthermore, MDSCs actively reinforce immune tolerance by inducing the differentiation of regulatory T cells (Tregs) and guiding local tissue macrophages toward an anti-inflammatory, tissue-reparative M2 phenotype [21,22,23].

Characterizing and isolating human MDSCs remains methodologically challenging due to the absence of a unique, lineage-specific surface marker. Currently, human MDSCs are broadly identified by the coexpression of the myeloid antigens CD11b and CD33, coupled with a characteristically low or negative expression of the major histocompatibility complex class II antigen, human leukocyte antigen D-related (${\text{HLA-DR}}^{\mathrm{low}/-}$) [24,25]. Based on surface marker landscapes, human MDSCs are stratified into two primary sub-populations: monocytic MDSCs (M-MDSCs), defined phenotypically as ${\mathrm{CD}11b}^{+}{\mathrm{CD}33}^{+}{\mathrm{CD}14}^{+}{\text{HLA-DR}}^{\mathrm{low}/-}$, and granulocytic or polymorphonuclear MDSCs (G-MDSCs or PMN-MDSCs), defined phenotypically as ${\mathrm{CD}11b}^{+}{\mathrm{CD}33}^{+}{\mathrm{CD}15}^{+}{\text{HLA-DR}}^{\mathrm{low}/-}$ (or alternatively characterized via CD66b expression) [25,26]. However, these surface marker combinations can overlap with activated classical monocytes, degranulating neutrophils, or immature myeloid precursors lacking immunomodulatory activity. Therefore, a clear distinction must be maintained between phenotypically defined MDSC-like cells (identified solely by surface markers) and functionally validated MDSCs (confirmed to suppress T-cell proliferation or cytokine production in vitro). In conditions such as obesity, type 2 diabetes, coronary artery disease, and heart failure, severe metabolic stress can disrupt myeloid cell function—causing cell populations to expand numerically while losing their suppressive activity. Relying solely on surface immunophenotyping without functional validation can lead to misinterpreting dysfunctional myeloid expansion as active immunosuppression in human clinical cohorts.

**Figure 1:**
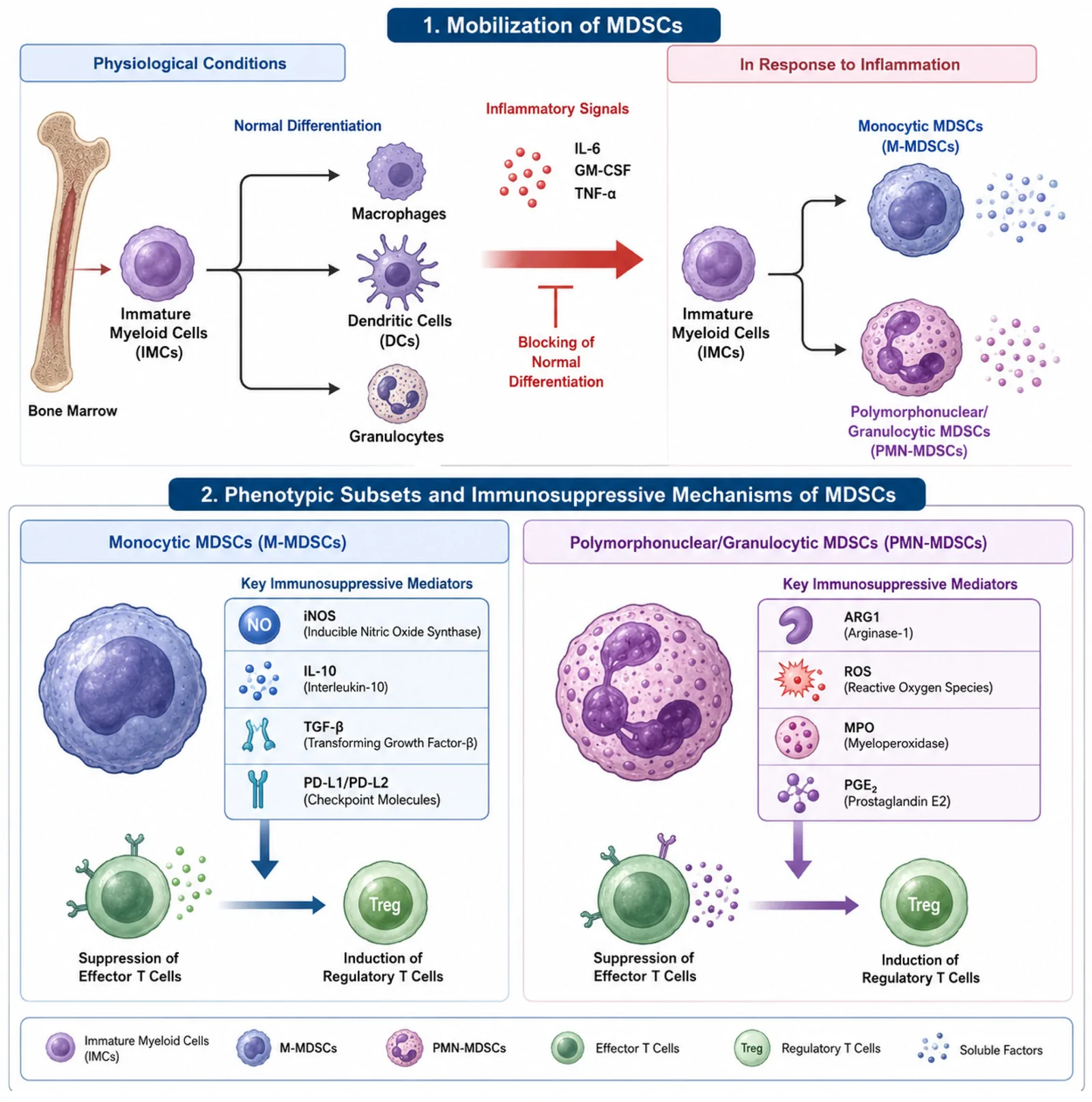
Mobilization, phenotypic subsets, and immunosuppressive mechanisms of myeloid-derived suppressor cells (MDSCs). Under physiological conditions, bone marrow immature myeloid cells (IMCs) differentiate into mature macrophages, dendritic cells (DCs), or granulocytes. In response to inflammatory signals (e.g., IL-6, GM-CSF, TNF-alpha), normal differentiation is blocked, leading to the mobilization of monocytic MDSCs (M-MDSCs) and polymorphonuclear/granulocytic MDSCs (G-MDSCs). M-MDSCs predominantly utilize inducible nitric oxide synthase (iNOS), interleukin-10 (IL-10), transforming growth factor-beta (TGF-beta), and PD-L1/PD-L2 checkpoints to suppress effector T cells and induce Tregs. G-MDSCs rely primarily on arginase-1 (ARG1), reactive oxygen species (ROS), myeloperoxidase (MPO), and prostaglandin E2 (PGE_2_) to exert immune suppression. Created with Banana Pie software using the GPT Image 2 model, https://www.bananapie.ai (accessed on 10 August 2026).

Mechanistically, M-MDSCs and G-MDSCs implement distinct yet complementary biochemical tools to quench hyperactive inflammatory responses. M-MDSCs utilize the upregulation of inducible nitric oxide synthase (iNOS) to generate high quantities of nitric oxide (NO) alongside the robust secretion of classic anti-inflammatory cytokines like IL-10 and TGF-β. In contrast, G-MDSCs primarily rely on the heightened production of intracellular reactive oxygen species (ROS), the release of arginase-1 (ARG1)—which depletes local L-arginine supplies to arrest T-cell proliferation—and the production of prostaglandin E2 (${\mathrm{PGE}}_{2}$) [10,27,28,29]. Furthermore, both major subsets are fully capable of directly disabling antigen-specific T-cell function through cell-surface immune checkpoints, binding to programmed cell death protein 1 (PD-1) expressed on lymphocytes via their upregulated ligands, PD-L1 and PD-L2. The dynamic orchestration of these biochemical pathways is heavily contingent upon the underlying disease stage, glycemic stress levels, and localized tissue microenvironments [30,31,32]. Figure 1 summarizes the phenotypic subsets of MDSCs as well as their recruitment and immunosuppressive mechanisms (Figure 1).

## 2. MDSCs in Obesity and Diabetes-Associated Metabolic Inflammation

### 2.1. Obesity and Adipose Tissue Homeostasis

Obesity serves as a foundational driver of systemic cardiometabolic risk, establishing a state of chronic, low-grade adipose tissue inflammation that alters resident immune populations [5,33,34]. Nutrient excess promotes adipocyte hypertrophy and hypoxia, triggering an increased infiltration of pro-inflammatory M1-polarized macrophages, dendritic cells, and $CD8^{+}$ cytotoxic T lymphocytes into the stromal vascular fraction [35,36,37]. In response to this expanding inflammatory state, MDSCs accumulate within peripheral reservoirs, specifically the liver and visceral adipose tissue blocks, to maintain immunological homeostasis [38,39].

Preclinical studies in high-fat diet (HFD) murine models show that murine MDSCs accumulate in large quantities to counter localized tissue stress. These cells suppress pro-inflammatory immune effectors, notably restricting the activation of cytotoxic $CD8^{+}$ T cells and shifting resident adipose tissue macrophages from an inflammatory M1 phenotype toward a protective, anti-inflammatory M2 state, which improves overall systemic insulin sensitivity [7,40,41,42]. This recruitment and functional activation are driven by upstream obesity-associated mediators, including leptin, interleukin-6 (IL-6), tumor necrosis factor-alpha (TNF-α), ${\mathrm{PGE}}_{2}$, and fatty acid synthase (FASN). Pharmacological interventions designed to stabilize metabolic health, such as the use of rapamycin (a mechanistic target of rapamycin complex 1 (mTORC1) inhibitor), expand endogenous regulatory immune populations, including MDSCs, to preserve systemic metabolic balance under lipid stress [43,44,45,46].

However, the expansion of MDSCs in obesity is highly nuanced and context dependent. In chronic high-fat diet settings, two distinct subsets of M-MDSCs with opposing biological trajectories have been observed: pro-inflammatory M-MDSCs (${\mathrm{CD}11b}^{+}{\mathrm{Ly}6G}^{-}{\mathrm{Ly}6C}^{\mathrm{high}}$) and reparative M-MDSCs (${\mathrm{CD}11b}^{+}{\mathrm{Ly}6G}^{-}{\mathrm{Ly}6C}^{\mathrm{low}}$) [47,48]. Visceral adipose tissue upregulates the chemokine C-C motif chemokine ligand 5 (CCL5), which signals through C-C motif chemokine receptor 5 (CCR5) to selectively recruit pro-inflammatory M-MDSCs. This subset-specific infiltration worsens local tissue inflammation and exacerbates insulin resistance, indicating that persistent metabolic overnutrition can skew MDSC function from protective immunoregulation toward maladaptive tissue dysfunction [49,50,51].

### 2.2. Type 1 Diabetes (T1D)

In type 1 diabetes (T1D), the primary pathophysiological event is the T-cell-mediated autoimmune destruction of pancreatic β-cells, characterized by intense peri-islet lymphocytic infiltration (insulitis). Given their established immunosuppressive capacity, MDSCs have become a major target for therapeutic optimization in T1D [52,53,54,55,56].

In autoimmune non-obese diabetic (NOD) mouse models, the adoptive transfer of functional MDSCs successfully prevents disease onset and significantly decreases lymphocyte infiltration within pancreatic islets. Similarly, the administration of MDSCs expanded ex vivo through a targeted cytokine cocktail consisting of granulocyte-macrophage colony-stimulating factor (GM-CSF), IL-6, and interleukin-1 beta (IL-1beta) attenuates systemic hyperglycemia and prolongs diabetes-free survival [56,57]. Furthermore, triggering endogenous MDSCs via an agonistic anti-TLR4/MD2 monoclonal antibody effectively reverses acute, newly established T1D by restoring peripheral immune tolerance [58,59]. Human clinical studies support these preclinical observations, showing a significant accumulation of M-MDSCs in the peripheral circulation of T1D patients and their genetically predisposed, high-risk first-degree relatives [60]. These circulating cells demonstrate a robust capacity to suppress autologous T-cell proliferation in a contact-dependent manner, highlighting their role as an endogenous checkpoint against autoimmune destruction [61].

While T2D involves metabolic inflammation and insulin resistance, T1D stems from autoimmune beta-cell destruction. Therefore, this autoimmune checkpoint function contrasts with the metabolically altered MDSCs seen in chronic T2D. Therefore, T1D may be considered as a mechanistic baseline control that contrasts pure autoimmune inflammation with chronic metabolic inflammation (T2D).

### 2.3. Type 2 Diabetes (T2D)

Type 2 diabetes (T2D) is defined by a combination of insulin resistance and chronic systemic inflammation [62]. Parallel to patterns observed in simple obesity, MDSC frequencies in peripheral organs and circulating blood rise substantially in both murine T2D models and human clinical cohorts, acting as an endogenous compensatory mechanism to limit chronic tissue damage [63]. In diabetic mouse models, the experimental depletion of ${\mathrm{Gr}1}^{+}$ myeloid suppressive cells severely worsens systemic inflammation, glucose intolerance, and peripheral insulin resistance, while expanding this cell population significantly improves these metabolic parameters [38,63].

Clinical investigations reveal that total cell counts do not fully reflect the complexities of the diabetic immune state, as subset composition and glycemic control heavily dictate patient outcomes. For example, patients with T2D exhibit a marked reduction in highly specific, protective regulatory sub-populations, such as ${\text{PD-L1}}^{+}$ G-MDSCs, ${\text{PD-L2}}^{+}$ M-MDSCs, and ${\text{PD-L2}}^{+}$ G-MDSCs, pointing to a selective loss of key immune checkpoint barriers despite an increase in total cell counts [64]. Furthermore, the degree of glycemic control directly correlates with circulating MDSC dynamics. Patients with poor glycemic control exhibit significantly higher frequencies of M-MDSCs that produce IL-10 and TGF-β compared to well-controlled diabetic subjects or healthy controls. This pronounced systemic expansion in decompensated patients can lead to excessive, non-localized immunosuppression, which may increase susceptibility to opportunistic infections or facilitate tumor immune evasion [65,66].

## 3. MDSCs in Cardiovascular Pathophysiology

### 3.1. Atherosclerosis and Lesion Biology

Cardiovascular disease in patients with obesity-associated diabetes is driven by sustained endothelial injury and vascular wall inflammation. Traditional risk factors do not fully account for this elevated risk, indicating that innate and adaptive immune cell interactions within the vascular intima are major contributors. In this context, MDSCs are recruited to the vascular wall where they act primarily as protective, anti-atherogenic agents [67,68,69].

Preclinical studies in atherosclerosis-prone ${\mathrm{LDLr}}^{-/-}$ or ${\mathrm{ApoE}}^{-/-}$ mouse models demonstrate that the adoptive transfer of ${\mathrm{CD}11b}^{+}{\mathrm{Gr}1}^{+}$ MDSCs reduces aortic lesion burden by 35% and lowers adventitial T-cell infiltration by 54%. This atheroprotective effect is mediated by the robust suppression of pro-inflammatory type 1 helper T (Th1) and type 17 helper T (Th17) vascular responses [69].

In early atherogenesis, the homing of protective M-MDSCs and regulatory T cells (Tregs) to dysfunctional endothelium is regulated by the CCR5-CCL5 chemokine axis. Experimental blockade of CCR5 alters plaque morphology, shifting it toward a stable phenotype characterized by increased smooth muscle cell coverage and a lower density of pro-inflammatory macrophages. Furthermore, certain anti-atherosclerotic therapies act partly through this pathway; for instance, the oral administration of heat shock protein 60 (HSP60) expands protective splenic and circulating M-MDSCs, shrinking overall plaque volume [70,71,72]. Similarly, glucagon-like peptide-1 (GLP-1) receptor agonists like exendin-4, alongside heme oxygenase-1 (HO-1) inducers such as hemin and bilirubin, reduce atherogenic risk factors partly by expanding endogenous bone marrow and blood MDSC pools [73,74].

This protective effect of MDSCs in the plaque versus the detrimental effect in visceral adipose tissue promoted by the same signal may be explained because the outcome of chemokine recruitment depends on the underlying tissue parenchyma. In visceral adipose tissue, CCL5/CCR5 signaling recruits Ly6C^mon^ M-MDSCs that aggravate local insulin resistance. In the vascular wall, CCL5/CCR5-mediated homing directs M-MDSCs to suppress local Th1/Th17 effector responses, slowing plaque progression. Moreover, high-fat diets and sustained hyperglycemia can induce cellular metabolic reprogramming. This elevated intracellular glycolysis can drive MDSCs to trans-differentiate into mature pro-inflammatory macrophages, altering their suppressive phenotype.

### 3.2. Coronary Artery Disease (CAD) and Acute Coronary Syndrome (ACS)

In clinical coronary artery disease (CAD), a systemic imbalance between MDSC sub-populations frequently tracks with disease severity. Patients with established CAD show an increase in circulating M-MDSCs accompanied by a simultaneous reduction in G-MDSCs, indicating that coronary atherosclerosis alters bone marrow myeloid output [75,76,77].

In acute coronary syndrome (ACS), characterized by plaque rupture and acute myocardial ischemia, the mobilization of ${\mathrm{CD}14}^{+}{\text{HLA-DR}}^{\mathrm{low}/-}$ M-MDSCs increases sharply in human clinical cohorts. Elevated levels of these circulating M-MDSCs correlate with the presence of multivessel disease, increased coronary lesion burden, severe left ventricular dysfunction, and obesity. Rather than driving vascular injury, this systemic surge represents an endogenous immune effort to suppress ischemia-induced myocardial inflammation and promote tissue healing, making circulating M-MDSC counts a possible useful biomarker for evaluating clinical severity and patient response to therapy [75,78].

**Figure 2:**
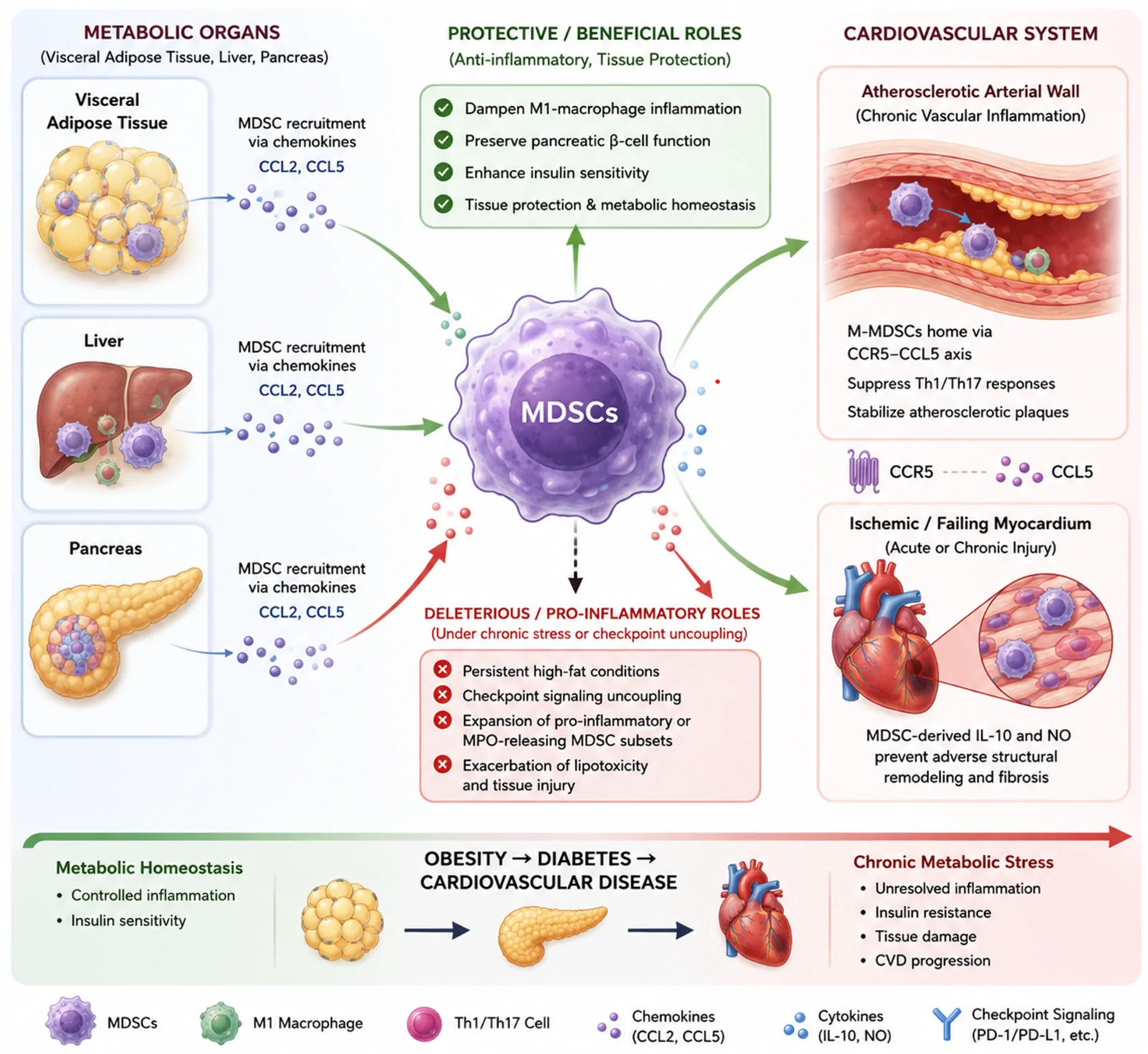
Cross-talk and context-dependent dual roles of MDSCs across the obesity-diabetes-cardiovascular disease continuum. In metabolic organs (visceral adipose tissue, liver, pancreas), MDSCs are recruited via chemokines (CCL2, CCL5) to dampen M1-macrophage inflammation, preserve pancreatic *β*-cell function, and enhance insulin sensitivity. However, under persistent high-fat conditions or uncoupled checkpoint signaling, pro-inflammatory or MPO-releasing subsets exacerbate lipotoxicity and tissue injury. In the cardiovascular system, M-MDSCs home to arterial walls via CCR5-CCL5 to suppress Th1/Th17-driven vascular inflammation and stabilize atherosclerotic plaques, while in the ischemic or failing myocardium, MDSC-derived IL-10 and NO prevent adverse structural remodeling. Created with Banana Pie software using the GPT Image 2 model, https://www.bananapie.ai (accessed on 10 August 2026).

Figure 2 summarizes the dual role of MDSCs in the obesity-associated cardiovascular risk in diabetes.

### 3.3. Heart Failure and Myocardial Remodeling

Heart failure (HF) involves complex interactions between innate and adaptive immune pathways, where chronic cytokine stress from IL-1, IL-6, and TNF-α drives adverse myocardial remodeling and progressive ventricular dilation. Circulating MDSCs in HF patients increase in proportion to disease severity, correlating with elevated plasma concentrations of IL-6, IL-10, and TGF-β [79,80,81,82].

Preclinical loss-of-function models demonstrate that the pharmaceutical depletion of endogenous MDSCs accelerates pathological cardiac remodeling and exacerbates myocardial inflammation. Conversely, the adoptive transfer or targeted expansion of MDSCs rescues ventricular function, protecting the myocardium through mechanisms dependent on IL-10 release and iNOS-derived NO production [79,83,84].

Concurrently, treatment with rapamycin increases endogenous MDSC frequencies by inhibiting their differentiation into mature, pro-inflammatory myeloid effectors, alleviating heart failure progression. This cardioprotective profile extends across the vascular network; in experimental models of aortic dissection, MDSC depletion accelerates aortic wall degradation and rupture, confirming their vital role in preserving macrovascular structural integrity under mechanical and inflammatory stress [79,85].

Nevertheless, it should be taken into account that preclinical mouse models utilize uniform markers and allow direct causal testing via adoptive transfer or cell depletion, whereas human studies rely on multi-color flow cytometry in clinical cohorts, where circulating cell frequencies reflect associational data rather than proven causation. Moreover, flow cytometric identification of surface markers alone does not guarantee active T-cell suppression. In severe obesity and advanced T2D, expanded MDSC-like populations can lose functional suppressive capacity, suggesting that surface marker presence can become uncoupled from true immunoregulatory function.

## 4. Clinical Biomarker Patterns, Functional Heterogeneity, and Risks

In patients with obesity and metabolic syndrome, circulating MDSC frequencies correlate closely with established inflammatory and cardiometabolic risk markers, including elevated serum triglycerides, fasting glucose, glycated hemoglobin (HbA1c), high-sensitivity C-reactive protein (hsCRP), increased waist circumference, and depressed high-density lipoprotein (HDL) cholesterol. For example, overweight and obese men exhibit an approximate 3.5-fold increase in circulating M-MDSCs compared to lean counterparts, while T2D cohorts consistently present with elevated fractions of suppressive ${\mathrm{CD}33}^{+}{\text{HLA-DR}}^{\mathrm{low}}$ cells [38,86,87].

However, a critical clinical caveat rests on the profound functional heterogeneity of these cells. Surface immunophenotyping alone does not guarantee uniform biological suppressive capacity. In some individuals with severe obesity, expanded populations of MDSC-like cells exhibit weak T-cell suppressive capacity in vitro, demonstrating that metabolic stress can uncouple surface marker expression from true immunoregulatory function [38].

Furthermore, persistent MDSC expansion in certain non-diabetic chronic inflammatory states can predict adverse outcomes. In patients undergoing chronic hemodialysis, persistently elevated circulating M-MDSCs independently predict future ischemic strokes and acute heart failure events. Under these conditions, M-MDSCs acquire an altered phenotype that promotes trans-endothelial migration and rapid recruitment into stable plaques, accelerating atherogenic transformation. Therefore, MDSCs are best understood as context-dependent immunoregulators rather than simple markers of risk, as their phenotypic balance and functional quality dictate whether they protect or injure the cardiovascular system [79,88,89].

Moreover, despite their biological relevance, the clinical applicability of MDSCs remains limited by the lack of standardized phenotyping, validated cut-offs, and prospective validation. In addition, their incremental prognostic value beyond established cardiovascular biomarkers has not yet been demonstrated. Accordingly, MDSCs should currently be regarded as promising research biomarkers rather than routine clinical tools.

## 5. Therapeutic Horizons and Safety Challenges

Harnessing MDSCs for clinical utility in metabolic and cardiovascular settings offers several promising pathways. Ex vivo cellular propagation techniques—such as treating autologous myeloid progenitors with defined cytokine combinations including IL-6, GM-CSF, and IL-1—have successfully generated stable, immunoregulatory MDSCs that prolong survival and minimize microvascular and macrovascular complications in diabetic mice. Additionally, a second approach may be the use of previous cardiometabolic drugs that may modulate MDSCs. Thus, small-molecule agents like rapamycin, GLP-1 receptor agonists, and specific HO-1 inducers provide reproducible methods to augment endogenous MDSC networks without requiring cell transplantation [68,90]. Nevertheless, to date, no clinical trials have evaluated MDSC-targeted therapies in human obesity, T2D, or CVD.

In any case, despite these therapeutic opportunities, several major safety challenges must be resolved before clinical translation. Thus, sustained, non-localized systemic immunosuppression can compromise host immune defense, increasing the risk of opportunistic infections and limiting the host’s capacity to clear pathogens. Moreover, in oncology, tumor-derived MDSCs are well-known drivers of malignant progression, neo-angiogenesis, and resistance to immune checkpoint therapies. Expanding MDSCs to treat cardiometabolic diseases carries the risk of inadvertently supporting occult malignancies by creating a permissive, immunosuppressive environment that allows tumor cells to evade T-cell and NK-cell surveillance [14]. In addition, MDSCs are highly sensitive to microenvironmental changes. For example, in acute ischemic stroke models, infiltrated MDSCs initially limit acute brain injury but rapidly differentiate into mature pro-inflammatory cells as the tissue microenvironment changes [91].

To overcome these barriers and safely implement MDSC-based therapies, future research must utilize advanced multi-omic single-cell profiling and spatial cell-tagging techniques. These methodologies will clarify the temporal and spatial distribution of MDSCs within tissues, enabling the development of targeted approaches to selectively modulate protective subsets without triggering off-target immune suppression or lineage destabilization. In addition, establishing safe therapeutic strategies will depend on developing localized delivery systems—such as targeted nanoparticles or tissue-specific chemokine modulators—capable of harnessing the anti-inflammatory benefits of MDSCs within injured arteries or myocardium without inducing systemic immunosuppression or oncological risk.

On the other hand, no clinical trials have evaluated MDSC-targeted strategies in obesity, diabetes, or cardiovascular disease. Whether current cardiometabolic therapies, such as GLP-1 receptor agonists, SGLT2 inhibitors, or statins, exert part of their benefits through modulation of MDSC biology also warrants further investigation.

## 6. Conclusions and Outstanding Questions

MDSCs represent key, context-dependent cellular regulators operating at the intersection of obesity, diabetes, and cardiovascular disease. They challenge basic disease models by demonstrating a dual capacity: they can protect vascular tissues by suppressing Th1/Th17 driven plaque progression, stabilizing matrix architecture in heart failure, and dampening visceral adipose tissue inflammation. Concurrently, persistent sub-population imbalances and uncoupled functional suppression under glycemic and lipotoxic stress can worsen local tissue injury or indicate advanced clinical disease.

To advance this field toward safe clinical application, several core questions must be addressed: what specific molecular pathways govern the immunosuppressive quality of distinct human MDSC subsets within metabolic and cardiovascular tissues? Which upstream signaling networks control the operational differentiation and phenotypic stability of MDSCs within lipid-rich, hyper-glycemic tissue environments? How can clinical therapies be engineered to selectively exploit the anti-inflammatory benefits of MDSCs within the vascular wall or myocardium without inducing systemic immunodeficiency or promoting oncological immune evasion?

Resolving these mechanistic questions will be essential for transforming our understanding of MDSC biology into precise, safe, and effective therapeutic strategies for patients with cardiometabolic disease.

Moreover, even though MDSCs represent a promising pathophysiological mechanism and potential research biomarkers, their clinical implementation will require methodological standardization, prospective validation, and evidence of incremental value beyond established cardiovascular biomarkers.

## Data Availability

No new data were created or analyzed in this study. Data sharing is not applicable to this article.
